# Schisandrin B: A Double-Edged Sword in Nonalcoholic Fatty Liver Disease

**DOI:** 10.1155/2016/6171658

**Published:** 2016-10-26

**Authors:** Pou Kuan Leong, Kam Ming Ko

**Affiliations:** Division of Life Science, The Hong Kong University of Science and Technology, Clear Water Bay, Hong Kong

## Abstract

Nonalcoholic fatty liver disease (NAFLD) is a spectrum of liver lesions ranging from hepatic steatosis, nonalcoholic steatohepatitis, hepatic cirrhosis, and hepatocellular carcinoma. The high global prevalence of NAFLD has underlined the important public health implications of this disease. The pathogenesis of NAFLD involves the abnormal accumulation of free fatty acids, oxidative stress, endoplasmic reticulum (ER) stress, and a proinflammatory state in the liver. Schisandrin B (Sch B), an active dibenzooctadiene lignan isolated from the fruit of* Schisandra chinensis* (a traditional Chinese herb), was found to possess antihyperlipidemic, antioxidant, anti-ER stress, and anti-inflammatory activities in cultured hepatocytes* in vitro* and in rodent livers* in vivo*. Whereas a long-term, low dose regimen of Sch B induces an antihyperlipidemic response in obese mice fed a high fat diet, a single bolus high dose of Sch B increases serum/hepatic lipid levels in mice. This differential action of Sch B is likely related to a dose/time-dependent biphasic response on lipid metabolism in mice. The hepatoprotection afforded by Sch B against oxidative stress, ER stress, and inflammation has been widely reported. The ensemble of results suggests that Sch B may offer potential as a therapeutic agent for NAFLD. The optimal dose and duration of Sch B treatment need to be established in order to ensure maximal efficacy and safety when used in humans.

## 1. Introduction

Nonalcoholic fatty liver disease (NAFLD) constitutes a spectrum of alcohol consumption-independent liver lesions ranging from hepatic steatosis, nonalcoholic steatohepatitis (NASH), hepatic cirrhosis, and hepatocellular carcinoma (HCC) [1]. Recently, an epidemiological meta-analysis study has shown that the global prevalence of NAFLD is 25% [2], indicative of the magnitude of the clinical as well as the economic burden globally. To cope with this situation, preventative interventions are urgently needed. Traditional Chinese medicinal herbs, which have a long history of use in safeguarding health, may offer a promising approach for the prevention and/or treatment of NAFLD. In this review, we will consider the pathogenesis of NAFLD, followed by a discussion of the hepatoprotective action of schisandrin B (Sch B), an active dibenzooctadiene lignan isolated from the fruit of* Schisandra chinensis* (FSC, a traditional Chinese herb), in relation to the pathogenesis of NAFLD.

## 2. A Brief Introduction to the Pathogenesis of NAFLD

**Figure 1:**
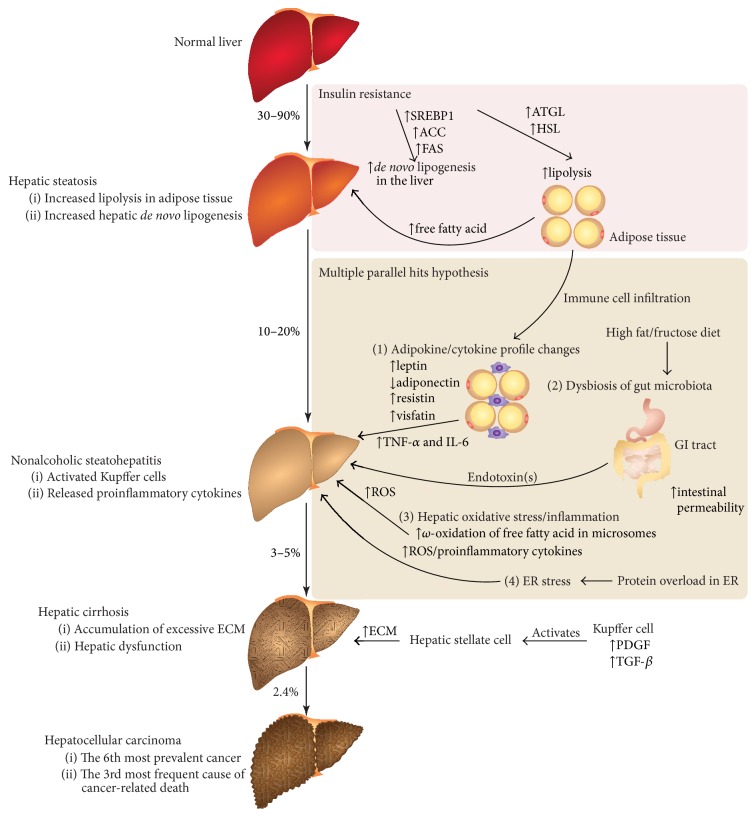
The pathogenesis of nonalcoholic fatty liver disease (NAFLD). The percentage shown along the arrow indicates the prevalence of the pathogenesis leading to the next stage of NAFLD. Hallmarks and important features of each stage of NAFLD are indicated. Insulin resistance is an important pathological factor for the development of hepatic steatosis (a benign stage of NAFLD), presumably due to the induction of hepatic* de novo* lipogenesis as well as lipolysis of adipose tissue. Key enzymes involved in these processes are indicated (in pink box). Changes in the profile of adipokines and cytokine, dysbiosis of gut microbiota, hepatic oxidative stress/inflammation, and endoplasmic reticulum ER stress are regarded as the “multiple parallel hits” of the pathogenesis of NASH from hepatic steatosis. Key factors involved in the pathogenesis are indicated (in orange box). SREBP1: sterol regulatory element-binding protein-1; ACC: acetyl-CoA carboxylase; FAS: fatty acid synthase; ATGL: adipose triglyceride lipase; HSL: hormone-sensitive lipase; GI tract: gastrointestinal tract; TNF-*α*: tumor necrosis factor-*α*; IL-6: interleukin-6; ROS: reactive oxygen species; ER: endoplasmic reticulum; PDGF: platelet-derived growth factor; TGF-*β*: transforming growth factor-*β*; ECM: extracellular matrix.

Obesity, as well as the associated insulin resistance, is a predisposing factor in the pathogenesis of NAFLD. About 30–90% of obese individuals will eventually develop hepatic steatosis, which is defined as an abnormal accumulation of lipid at ≥5% of the organ weight [3]. However, 10–20% of patients with hepatic steatosis may go on to develop NASH in which inflammation and hepatic tissue damage occur [4]. Patients (3–5%), with NASH, in whom livers exhibit a repeated damage-and-repair cycle due to chronic inflammation, may go on to develop cirrhosis [1]. Cirrhosis, which is referred to as the dysfunction of fibrotic liver, is one of the risk factors for hepatocellular carcinoma [5] (Figure 1).

### 2.1. Hepatic Steatosis

The liver plays a pivotal role in the metabolic homeostasis of carbohydrates, lipids, and proteins. Hepatic lipid content is governed by the uptake of free fatty acids as well as the export of processed lipids [such as very low density lipoprotein (VLDL)] [6]. Free fatty acids in the liver can arise from the lipolysis of triacylglycerol (TAG) which is stored in white adipose tissue under fasting conditions. Another source of fatty acids is the dietary intake of lipid, which is processed into chylomicrons. The liver can also undergo* de novo *lipogenesis, in which excess blood glucose can be converted into TAG under postprandial conditions [6, 7]. In* de novo* lipogenesis, the major transcription factors, namely, sterol regulatory element-binding protein-1c (SREBP-1c) and carbohydrate-responsive element-binding protein (ChREBP), can induce the expression of an array of enzymes involved in lipogenesis (such as acetyl-CoA carboxylase (AAC) and fatty acid synthase (FAS)) in the presence of high levels of insulin and glucose [6, 7]. In this regard, insulin plays a critical role in the regulation of lipogenesis via the induction of SREBP-1c activity. On the other hand, lipid mitochondrial *β*-oxidation in the liver can also reduce TAG levels [6, 7]. In the process of mitochondrial *β*-oxidation, long-chain-fatty-acid-CoA ligase, carnitine palmitoyltransferase I (CPT1), and carnitine palmitoyltransferase 2 (CPT2) play critical roles in the transfer of free fatty acids into the mitochondrial matrix, with resultant production of fatty acyl-CoA which is the initial substrate for *β*-oxidation [6, 7]. The fatty acyl-CoA is metabolized into acetyl-CoA and water with the generation of ATP. In addition, hepatic lipids can also serve as an endogenous supply of lipid in the body. Hepatic triglycerides (TG), cholesterol, and apolipoproteins can be assembled into VLDLs which circulate in the bloodstream for the delivery of lipids to peripheral tissues [6].

The dysregulation of intermediary metabolism in the liver, which is usually observed in insulin-resistant/obese individuals, can lead to an abnormal accumulation of lipid in the liver [8]. This excessive accumulation predominantly arises from the overflow of free fatty acids following lipolysis in white adipose tissue as well as hepatic* de novo* lipogenesis. However, pathological changes in hepatic mitochondrial *β*-oxidation and the export of VLDLs are less likely to be involved [9]. Given that insulin regulates lipolysis in adipose tissue as well as* de novo* lipogenesis in the liver, insulin resistance is associated with hepatic steatosis [8]. In healthy individuals, insulin can exert a powerful antilipolytic action by the inactivation of hormone-sensitive lipase (HSL) via the phosphatidylinositol 3-kinase (PI3K)/Akt/phosphodiesterase 3B pathway [10]. In insulin-resistant individuals, the reduced sensitivity of white adipocytes to insulin can therefore lead to increased lipolysis [10]. In healthy individuals, insulin can suppress gluconeogenesis and induce lipogenesis in the liver. However, in the liver of rodents, the induction of insulin resistance is associated with overproduction of glucose (leading to hyperglycemia) as well as overinduction of SREBP-1c (which leads to increased hepatic* de novo* lipogenesis) [11, 12]. This paradoxical observation suggests differential regulation of hepatic gluconeogenesis and lipogenesis by insulin in insulin-resistant rodents, presumably due to diversity in insulin receptor signal transduction pathways [13]. The insulin receptor substrates IRS1 and IRS2 bind to the activated insulin receptor and serve as adaptor molecules for the further propagation of signal transduction. Shimomura et al. have hypothesized that insulin selectively suppresses IRS2 (which regulates gluconeogenic genes) but activates IRS1 (which induces lipogenic genes) [11], with resultant suppression of gluconeogenesis and enhancement of lipogenesis. An alternative hypothesis is that endoplasmic reticulum (ER) stress, during which excessive unfolded/misfolded proteins accumulate in the ER, can induce the expression of lipogenic genes in insulin-resistant liver, leading to the suppression of gluconeogenesis and enhancement of lipogenesis [14].

### 2.2. Nonalcoholic Steatohepatitis (NASH)

Given that only 10–20% of patients with HS will develop NASH, the pathogenesis of NASH was traditionally hypothesized to be a “two-hit” process [15], in which the “first hit” involves the excessive accumulation of lipid in the liver (i.e., hepatic steatosis) and the “second hit” involves risk factors (such as bacteria-derived endotoxin) that can induce liver inflammation [15]. Recently, accumulating experimental evidence has demonstrated the insufficiency of the “two-hit” hypothesis in explaining the complicated pathogenesis of NAFLD. The relatively low incidence rate (10–20%) of NASH in patients with hepatic steatosis suggests that hepatic steatosis is a benign state in the majority of NAFLD patients, while NASH is likely to be an optional diagnosis after fulfilling certain pathological conditions [16]. Tilg and Moschen have proposed a new model of the evolution of inflammation in NAFLD, namely, the “multiple parallel hits” hypothesis [16], in which the development of NASH from hepatic steatosis may involve many “hits,” including dysbiosis of the gut microbiota, the release of proinflammatory cytokines, hepatic oxidative stress/inflammation, pathological changes in the plasma level of adipokines, and genetic/epigenetic factors, acting in parallel, with resultant pathogenesis of NASH. In this section, we will mainly focus on the effects of gut microbiota, hepatic oxidative stress/inflammation, ER stress, and adipokines on the pathogenesis of NAFLD. Genetic and epigenetic factors in relation to NAFLD have been reviewed by Dongiovanni and Valenti [17].

#### 2.2.1. Dysbiosis of Gut Microbiota

The involvement of gut microbiota in the development of high fat diet-induced obesity in mice was first demonstrated by Turnbaugh et al., who demonstrated that the relative abundance of two gut bacterial populations, namely, Bacteroidetes and Firmicutes, is associated with the phenotype of obese and lean mice (and this was also seen in human volunteers) [18, 19]. This postulation was also supported by the experimental finding that the inoculation of germ-free mice with “obese microbiota” (isolated from high fat diet-fed mice) can lead to significant increases in body weight, total body fat disposition, hepatic lipogenesis, and insulin resistance relative to those animals transplanted with “lean microbiota” (isolated from control mice) [19, 20]. The ability of a high fat diet to decrease the gut bacterial ratio of Bacteroidetes to Firmicutes (i.e., the dysbiosis of gut microbiota) further confirms the interrelationships among diet, gut microbiota, and NAFLD phenotype [21, 22]. In addition to the feeding of a high fat diet, the effect of a high carbohydrate (particularly fructose) diet on NAFLD in relation to gut microbiota has also recently been described. Given that fructose is primarily metabolized in the liver, a diet with excessive fructose was found to increase the incidence of hepatic steatosis [23–25], presumably due to the dysbiosis of gut microbiota and the increase in the permeability of intestine to microbiome as well as increased levels of endotoxins in blood. The increased permeability of intestine to microbiome likely increases the risk of microbiome infection, with resultant endotoxemia [26, 27]. In response to endotoxemia, the residential macrophages in the liver (Kupffer cells) are activated* via* the toll-like receptor and subsequently release proinflammatory cytokines, which is a predisposing factor in the pathogenesis of NASH [28–30]. Under normal physiological conditions, mammalian nondigestible carbohydrates (such as dietary fibers) can be metabolized by gut microbiota, with resultant production of short-chain fatty acids (SCFA), which can maintain a favorable environment for microbial flora in the GI tract [31]. In this regard, the replacement of nondigestible carbohydrates with simple carbohydrates (such as fructose) was found to change the microbial composition of the gut, particularly the ratio between Bacteroidetes and Firmicutes [32]. The ensemble of experimental findings strongly suggests the interrelationships among diet, gut microbiota, and NAFLD. However, the direct linkages among diet, gut microbiota, and NAFLD remain to be investigated.

#### 2.2.2. Hepatic Inflammation and Oxidative Stress

Recent studies have indicated that saturated fatty acids can activate the proinflammatory toll-like receptor 4/transforming growth factor *β*-activated kinase-binding protein/c-Jun N-terminal kinase/nuclear factor-*κ*B (NF-*κ*B) signaling cascade, with the resultant release of interleukin-1*β* (IL-1*β*), tumor necrosis factor-*α* (TNF-*α*), interleukin-6 (IL-6), and transforming growth factor-*β* (TGF-*β*) [33]. The cytokines further induce the infiltration of inflammatory cells, leading to a vicious cycle that leads to the development of liver damage. The involvement of toll-like receptor 4 in the development of NASH was supported by a study showing that the feeding of a high fat diet did not induce obesity, insulin resistance, or inflammation in toll-like receptor 4 knockout mice [34]. In patients with hepatic steatosis, the increased availability of free fatty acids in the liver likely increases mitochondrial *β*-oxidation [35], with a resultant increase in the production of acetyl-CoA. The overproduction of acetyl-CoA can overwhelm the capacity of the tricarboxylic acid (TCA) cycle and the electron transfer chain (ETC) as well as the ATP synthase-catalyzed reaction, leading to increased leakage of electrons from ETC and hence the generation of reactive oxygen species (ROS) [36]. In addition, mitochondrial *β*-oxidation is the major mechanism for the hepatic disposition of free fatty acids under physiological conditions [35]. To cope with the hyperlipidemia in hepatic steatosis, peroxisomal and microsomal *ω*-oxidation of fatty acids are activated in a complementary fashion. Peroxisomal and microsomal oxidation of fatty acids involve cytochrome P_450_ 2E1 and cytochrome P_450_ reductase, both of which generate ROS as byproducts of the catalytic reaction [37, 38]. ROS can lead to the peroxidation of lipid molecules, which in turn can activate residential macrophages (Kupffer cells) in the liver and thereby increase the extent of inflammation.

#### 2.2.3. Endoplasmic Reticulum Stress

With a high metabolic rate, hepatocytes have a high capacity for protein synthesis and are enriched with ER for protein folding. Any pathological factors that perturb ER folding capacity (e.g., ER stress) can trigger an unfolding protein response, during which both the expression of chaperones and the ER-associated protein degradation are enhanced, with the amount of protein molecules entering the ER being reduced [39]. Recently, it has been demonstrated that the induction of biochemical factors involving a protein unfolding response is present in patients with NASH, suggesting the possible association of an ER stress/unfolding protein response in the pathogenesis of NASH [40]. Consistent with this postulation, ER stress was found to be associated with the aforementioned risk factors involving the “multiple parallel hits” hypothesis in the development of NASH. As such, the increase of protein load in the ER would increase the generation of ROS, presumably due to the activation of ER oxidoreductases, the enhanced electron flow in mitochondrial ETC, and the induction of NADPH oxidase [41]. In addition, ER stress can activate JNK/NF-*κ*B via the IRE1*α*-TRAF2 complex, leading to the release of proinflammatory cytokines [42]. The interaction of inflammation, oxidative stress, and ER stress in the pathogenesis of NASH remains to be elucidated.

#### 2.2.4. Adipokine/Cytokine Release from Adipose Tissue

In the last decade, the adipose tissue has been considered to be an endocrine tissue by the virtue of its ability to secrete adipokines, which can induce autocrine, paracrine, and endocrine functions relating to energy metabolism. As such, patients with obesity or other metabolic disorders were found to exhibit an abnormality in the secretion profile of adipokines [43]. In addition, the immune cell-infiltrating adipose tissue (particularly as found in obese individuals) as well as endothelial cells of adipose tissue was shown to secrete classical proinflammatory cytokines (such as TNF-*α* and IL-6). Given the association between obesity/insulin resistance and NAFLD [8], the role of adipokines/cytokine in relation to the pathogenesis of NAFLD has been emphasized recently [43]. The effects of adipokines (such as leptin [44–50], adiponectin [51–54], resistin [55–61], and visfatin [56, 62–64]) as well as proinflammatory cytokines (such as TNF-*α* and IL-6 [65–70]) arising from adipocytes and/or immune cell-infiltrating adipose tissue on the pathogenesis of NAFLD are summarized in Table 1. While adiponectin can serve as a protective adipokine in the pathogenesis of NAFLD, the high levels of leptin, resistin, visfatin, TNF-*α*, and IL-6 seem to be involved in the pathogenesis of NAFLD (Table 1).

### 2.3. Hepatic Cirrhosis

Hepatic steatosis and NASH are associated with the pathogenesis of hepatic fibrosis/cirrhosis. The development of hepatic fibrosis, during which hepatic parenchymal cells undergo regeneration for the replacement of necrotic/apoptotic cells, is a natural wound-healing process in response to liver injury [71]. This wound-healing process is accompanied by an inflammatory response as well as accumulation of newly synthesized extracellular matrix (ECM). Under conditions of repeated and persistent hepatic damage, Kupffer cells are activated and release platelet-derived growth factor (PDGF) that stimulates the proliferation of hepatic stellate cells (HSC), which are the major ECM producing cells in the liver [72]. The activated HSC may differentiate into myofibroblast-like cells that are proinflammatory and fibrogenic. The release of proinflammatory cytokines from HSC potentiates the inflammatory response, leading to a vicious cycle of tissue injury [72]. Hepatic cirrhosis is a pathological condition in which metabolic functions of the liver are suppressed by the excessive disposition of ECM arising from activated HSC. Despite the fact that the precise mechanism underlying NAFLD-mediated hepatic cirrhosis has yet to be elucidated, redox-regulated hepatic fibrosis is likely involved in its pathogenesis [73]. ROS, presumably arising during the development of NASH, are hypothesized to induce the release of TGF-*β*, which is one of the principal cytokines in the pathogenesis of human hepatic fibrosis. In this regard, TGF-*β* can stimulate the differentiation of HSC into myofibroblast-like cells as well as the associated production of ECM and the inhibition of ECM degradation [72]. Angiotensin II, a vasoactive cytokine, was found to promote fibrogenesis in activated HSC [74].

### 2.4. Hepatocellular Carcinoma (HCC)

Recently, a number of meta-analyses regarding the prevalence of HCC have indicated that the cumulative mortality from HCC is 0–3% in patients who are not in the cirrhotic stage, which includes hepatic steatosis as well as NASH (for a study period of up to 20 years), whereas the cumulative incidence of mortality in patients with cirrhotic NASH ranges from approximately 2% over 7 years to approximately 13% over 3 years [83]. This observation suggests that the presence of cirrhosis may predispose to the development of HCC. Inflammation, diabetes, and obesity are hypothesized to be systemic risk factors for the pathogenesis of HCC. Epigenetic changes as well as mutations in genes that regulate hepatic growth and regeneration can induce “replicative immortality” (which refers to the ability of a cell population to proliferate continuously) in hepatocytes [5]. In addition to the altered receptor signaling arising from accumulated mutations in “immortal” hepatocytes, hyperinsulinemia as well as proinflammatory signaling can further amplify the signal for cell growth and proliferation, with a resultant increased risk of HCC [5].

## 3. Hepatoprotective Action of Sch B against NAFLD

The use of nutraceuticals, particularly with antioxidant, anti-ER stress, and anti-inflammatory activities, has been utilized as one of the approaches for the treatment of NAFLD [84]. Sch B is the most abundant dibenzocyclooctadiene lignan isolated from the FSC. In the realm of traditional Chinese medicine theory, the FSC is prescribed for the treatment of viral/chemical-induced hepatitis [85]. This suggests the possible therapeutic application of Sch B in NAFLD. In this regard, accumulating experimental evidence has revealed that Sch B, the principal active ingredient found in the FSC, possesses antihyperlipidemic, antioxidant, anti-inflammatory, and anticarcinogenic activities demonstrable in cultured hepatocytes* in vitro* and rodent livers* in vivo* [75, 86–88]. As mentioned earlier, the progression from hepatic steatosis to NASH involves “multiple parallel hits” in the pathological process. In addition, NASH has been recognized as a pathological condition that can favor the development of the end-stage liver disease [89]. In this section, the hepatoprotective action afforded by Sch B in the pathogenesis of NAFLD will be discussed.

### 3.1. The Differential Effect of Sch B on Lipid Content in Blood and Liver in Relation to the “Benign State” of NAFLD Pathogenesis

As already mentioned, the excessive accumulation of free fatty acids in the liver is the primary event in the pathogenesis of NAFLD (i.e., the “benign state” of NAFLD). In this regard, the effect of Sch B on hepatic/plasma lipid contents has been extensively investigated. A recent study has demonstrated that Sch B dose-dependently suppresses free fatty acid-induced steatosis in cultured L02 hepatocytes, in part* via* the inhibition of adipose differentiation-related protein (ADRP) and SREBP-1 [75]. Paralleling the results obtained in the cell-based study, Pan et al. have shown that the administration of Sch B in mice (50–200 mg/kg/d) with cholesterol/bile salts (2/0.5 g/kg/d) for a period of 4 to 6 days reduces hepatic total cholesterol and TG in hypercholesterolemic mice [76]. In a recent study by Pan et al., a single bolus dose of Sch B (0.2 to 1.6 g/kg, given 24 h prior to sacrifice) was unexpectedly found to increase hepatic cholesterol and TG levels in control mice [77]. Recently, Pan et al. have developed a novel mouse model of combined hyperlipidemia associated with steatosis and liver injury involving the oral administration of a Sch B and cholesterol/bile salt mixture given as a single dose [78]. In this experimental model, the combination of Sch B/cholesterol/bile salt (1/2/0.5 g/kg) was found to increase serum TG and total cholesterol (TC) levels, hepatic TG and TC levels, and serum alanine/aspartate aminotransferase activities (the latter used as indicators of hepatic damage). The results obtained from studies by Pan et al. suggest a complicated mechanism underlying the differential effect of Sch B on hepatic lipid content in control* versus* hypercholesterolemic mice. To reconcile the differential observations obtained from a number of cell-based and animal-based studies, a recent study by Kwan et al. may provide a possible explanation for these seemingly conflicting findings [79]. Kwan et al. have investigated the effect of a single bolus dose of Sch B (0.8 g/kg) in nonfasting and fasting mice as well as the effect of long-term low dose Sch B (50 mg/kg/d × 14 days) in control and high fat diet- (HFD-) fed mice. Firstly, the single bolus dose of Sch B was found to increase plasma TG and total cholesterol as well as serum free fatty acid levels in fasting but not in nonfasting mice. Secondly, the long-term low dose treatment with Sch B was shown to reduce hepatic TG levels, FAS activity, levels of SREBP1 and TNF-*α*, and the extent of hepatic fibrosis in HFD-fed mice. The beneficial effect of the long-term low dose treatment with Sch B was also associated with increases in the levels of adipose triglyceride lipase and hormone-sensitive lipase in the adipose tissue of HFD-fed mice, all of which are indicative of an increase in lipolysis. In support of this, recent studies conducted by Pan et al. have also demonstrated that an aqueous extract of the pulp of FSC, an ethanol extract of the pulp of FSC, and the seed of FSC (all of which presumably contain lower concentrations of Sch B) were found to ameliorate serum/hepatic lipid profiles in control and hypercholesterolemic mice [90, 91].

In an effort to explore the feasibility of Sch B for use in patients with NAFLD, the human equivalent doses of Sch B in the aforementioned studies were estimated, based on a dose conversion equation from animals to humans [92]. We further estimated the equivalent amount of Sch B as well as air-dried FSC for human consumption. While the optimal daily dose of Sch B in humans has yet to be determined, the suggested dose of air-dried FSC for the adult human is 0.5–1.5 g twice daily [93], which is equivalent to 20–60 mg of Sch B per day [94]. With this notion in mind, the recommended high dose of air-dried FSC (equivalent of 60 mg of Sch B per day in humans) is much lower than the low dose that induces hyperlipidemia in mice (which is equivalent to 973 mg of Sch B per day in the human). This suggests that the administration of Sch B-containing FSC at the recommended dose is likely to be safe. Until now, the safety and therapeutic action of Sch B in patients with NAFLD have not been investigated.* Schisandra Plus* (or Wei-Kang-Su (WKS) in Chinese) is a commercially available health product comprised of Ginseng Radix, Ophiopogonis Radix, and Schisandrae Fructus. A recent study has demonstrated that the recommended dose of WKS in humans can induce hepatoprotective effects in CCl_4_- and ethanol-intoxicated rats [95, 96]. Taken together, the experimental results suggest that Sch B (given at low dose, long-term) has a great potential to be a therapeutic agent for use in patients with NAFLD. Since the estimation of the equivalent dose in the human is based on equations from an animal-human translation study [92], the optimal dose and duration of Sch B treatment need to be investigated in clinical studies in order to maximize efficacy and ensure safety.

### 3.2. Hepatoprotective Action of Sch B in Relation to the “Multiple Parallel Hits” Hypothesis of NAFLD

With regard to the attenuation of the “multiple parallel hits” hypothesis of NAFLD, Sch B possesses antioxidant, anti-inflammatory, anti-ER stress, and anticarcinogenic activity. Sch B induces a cellular/hepatic glutathione antioxidant response and protects against menadione-induced cytotoxicity in cultured AML12 hepatocytes [86], as well as hepatotoxicity in CCl_4_-treated mice [97]. CCl_4_ hepatotoxicity involves the release of TNF-*α*, nitric oxide, and TGF-*α*/*β* from Kupffer cells in the liver, resulting in the production of hepatic fibrosis [98]. The hepatoprotection against CCl_4_ toxicity afforded by Sch B in rats is associated with enhancement of glutathione regeneration capacity [99]. The glutathione antioxidant system is regulated by nuclear factor erythroid 2-related factor-2 (Nrf2), the principal transcriptional regulator of cellular antioxidant genes, which in turn binds to its corresponding element, namely, the antioxidant response element (ARE), on DNA [100]. A recent study has elucidated the cytoprotective mechanism underlying the Sch-B-induced glutathione antioxidant response in cultured hepatocytes [101]. Sch B is metabolized by cytochrome P_450_ with concomitant production of ROS [101]. The ROS then activate the redox-sensitive ERK/Nrf2/ARE signaling cascade, with a resultant expression of an array of glutathione-related enzymes. Reduced glutathione (GSH) and glutathione-related antioxidant enzymes can operate in concert to ameliorate oxidative stress [101]. Given that oxidative stress is one of the risk factors for the development of NAFLD, the antioxidant properties of Sch B may be involved in its salutary effect in retarding the progression of NAFLD (particularly in the development of NASH and hepatic fibrosis). With the cross talk between the Nrf2-mediated antioxidant signaling pathway and the NF-*κ*B-mediated proinflammatory signaling cascade [102], the induction of an antioxidant response may be protective by inhibiting proinflammatory factors. This postulate is supported by the observation that Sch B reduces the extent of inflammation in lipopolysaccharide-activated (LPS-activated) RAW264.7 macrophages [81], LPS/ATP-activated isolated peritoneal macrophages [87], and concanavalin A-activated isolated splenocytes [103], presumably* via* the induction of an Nrf2-mediated antioxidant response. With regard to the endotoxemia arising from the increased permeability of the intestine to gut microbiota in dysbiosis, the ability of Sch B to induce an anti-inflammatory response against LPS activation (LPS being a classical bacterial endotoxin) further strengthens its beneficial effect in NAFLD. In support of this, Song et al. have demonstrated that the daily intake of an aqueous extract of FSC (equivalent to 6.7 g dried FSC per day) for 12 weeks can modulate the composition of gut microbiota, which correlates well with some changes in various metabolic parameters (such as fat mass, ALT, AST, HDL, and fasting blood glucose) in obese women [104]. However, a tendency towards improvement in obesity-related parameters (such as waist circumference, body weight, body mass index, and fat mass) was observed in FSC-treated subjects, when compared with a placebo group [104]. A further clinical study is worthwhile to conduct in an effort to confirm the beneficial effect of aqueous extracts of FSC (as well as Sch B) in obese individuals. The ability of Sch B to ameliorate hepatic steatosis in cultured HepG2 hepatocytes and in C57BL/6 mouse liver in relation to the protection against ER stress has recently been reported [82]. Gomisin N (a stereoisomer of Sch B) was found to reduce the expression of ER stress markers (such as glucose-regulated protein-78, CCAAT/enhancer binding protein homolog protein, and X-box-binding protein-1), proinflammatory cytokines, and lipogenic enzymes and the level of TG in palmitate-challenged HepG2 cells [82]. Results from the cell-based study were further confirmed by an animal study, in which gomisin N was found to suppress the expression of ER stress markers and the levels of hepatic TG and TC in tunicamycin (an ER stress inducer)-injected mice [82]. In addition, Sch B was found to inhibit cell proliferation and induce apoptosis in cultured human hepatoma SMMC-7721 cells via a caspase-3-dependent pathway [88]. This finding suggests the possibility that Sch B may be effective in the treatment of hepatocarcinoma. However, the effect of Sch B (or a FSC extract) on the levels of adipokines in cultured adipocytes/obese animals has yet to be investigated.

## 4. Conclusion

**Figure 2:**
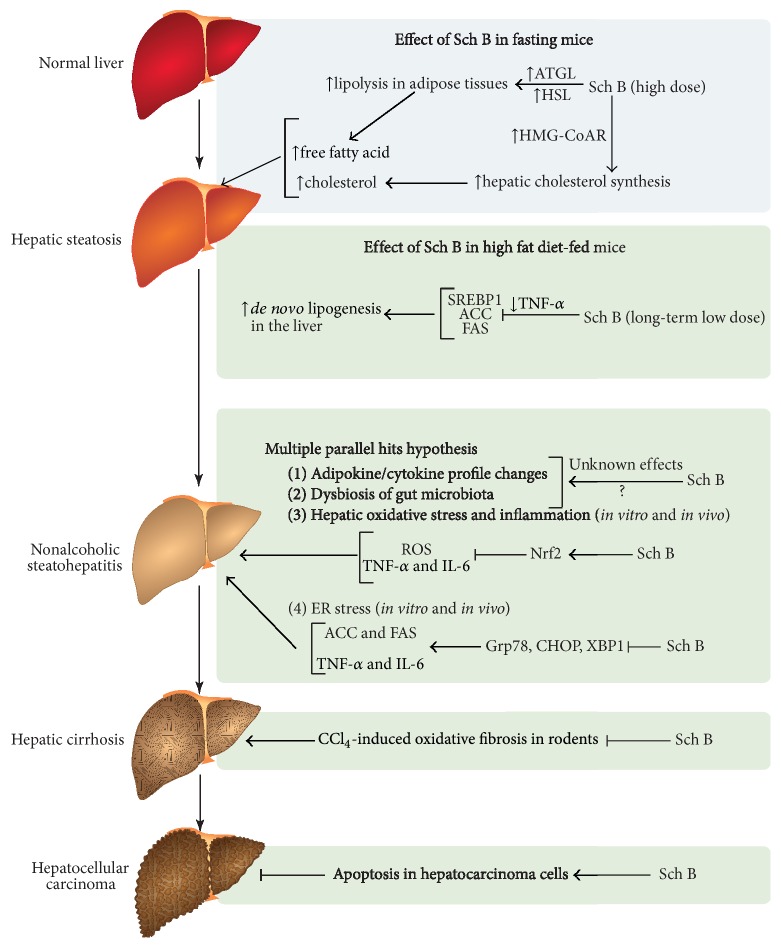
The hepatoprotection afforded by schisandrin B (Sch B) in relation to the pathogenesis of nonalcoholic fatty liver disease (NAFLD). The possible undesirable effect of Sch B is indicated in the blue box while the potential beneficial effects of Sch B are indicated in the green box. ATGL: adipose triglyceride lipase; HSL: hormone-sensitive lipase; HMG-CoAR: 3-hydroxy-3-methyl-glutaryl-coenzyme A reductase; TNF-*α*: tumor necrosis factor-*α*; SREBP1: sterol regulatory element-binding protein-1; ACC: acetyl-CoA carboxylase; FAS: fatty acid synthase; Nrf2: nuclear factor (erythroid-derived 2)-like 2; ROS: reactive oxygen species; IL-6: interleukin-6; Grp78: glucose-regulated protein-78; CHOP: CCAAT/enhancer binding protein homologous protein; XBP1: X-box-binding protein-1; CCl_4_: carbon tetrachloride.

The “multiple parallel hits” hypothesis for the pathogenesis of NAFLD suggests that obesity-associated hepatic steatosis is a “benign state,” which is followed by “multiple parallel hits” including dysbiosis of gut microbiota, release of adipokines/cytokines from adipose tissue, and hepatic oxidative stress and/or inflammation. The differential action of Sch B on lipid metabolism in mice is likely related to a dose/time-dependent biphasic response, with a long-term low dose of Sch B being beneficial in HFD-fed obese mice (Table 2). The hepatoprotection afforded by Sch B against oxidative stress, inflammation, and ER stress has also been widely reported (Table 2). The possible effect of Sch B on the modulation of gut microbiota in relation to the pathological factors of NAFLD is deserving of further investigation. Taken together, currently available experimental evidence strongly suggests that Sch B may provide a potentially effective intervention for the prevention and/or treatment of NAFLD (Figure 2).

## Figures and Tables

**Table 1:** Physiological/pathological effects of adipokines/cytokines in nonalcoholic fatty liver disease (NAFLD).

Adipokines/cytokines	Site of secretion	Normal physiological functions	Pathological effects in NAFLD	References
Leptin	White adipose tissue	Preserves the insulin sensitivity in the liver [48] (via the cross talk with the insulin signaling by activating the suppressor of cytokine signaling 3 [49])	Proinflammatory and fibrogenic [50] (via the activation of HSCs)	[48–50]

Adiponectin	Adipose tissue	Improves fatty acid metabolism by the induction of free fatty acid oxidation as well as inhibition of gluconeogenesis, free fatty acid uptake, and* de novo* lipogenesis in cultured hepatocytes [53]	The level of adiponectin is reduced in NAFLD [52]	[52–54]
	Injured hepatocytes	Suppresses the release of proinflammatory cytokines (TNF-*α* and IL-6) and induces the release of the anti-inflammatory cytokine (IL-10) in Kupffer cells [54]		
		Elicits an antifibrotic response by the inhibition of the release of TGF-*β* [54]		

Resistin	Macrophage-infiltrating adipose tissue [56]		Induces glucose intolerance and insulin resistance [55]	[55–61]
			Proinflammatory (the induction of release of TNF-*α*, IL-1*β*, IL-6, and IL-2 in resistin-incubated macrophages) [58–60]	
			Induces hepatic fibrosis (via the activation of HSCs and Kupffer cells [61] which release TGF-*β* and form collagen type 1)	

Visfatin	Macrophage-infiltrating adipose tissue [56]		Possesses nicotinamide phosphoribosyl-transferase activity, which is critical for the glucose-induced release of insulin in pancreatic beta cells* in vitro* and* in vivo* [63]	[56, 63, 64]
			Proinflammatory (the release of TNF-*α*, IL-6, and IL-1*β* from macrophages [64])	

TNF-*α* and IL-6	Adipocytes in individuals with insulin resistance or obesity [65]		Associated with the extent of obesity/adiposity in patients [66, 67]	[65–70]
			Distally influence the metabolic functions of liver via the JNK1-mediated release of IL-6 [68]	
			Facilitate the development of NASH and HCC (via the induction of oncogenic factor STAT3 and release of TNF-*α* and IL-6) [70]	

HSCs: hepatic satellite cells; TNF-*α*: tumor necrosis factor-*α*; IL-6: interleukin-6; IL-10: interleukin-10; TGF-*β*: transforming growth factor-*β*; IL-2: interleukin-2; IL-1*β*: interleukin-1*β*; JNK1: c-Jun N-terminal kinase-1; NASH: nonalcoholic steatohepatitis; HCC: hepatocellular carcinoma; STAT3: signal transducer and activator of transcription-3.

**Table 2:** Pharmacological effects of schisandrin B on nonalcoholic fatty liver disease (NAFLD).

Effects on NAFLD	Experimental models	Concentration/dose	Pharmacological actions	References
Modulatory effects on lipid contents	Free fatty acid-induced steatotic L02 hepatocytes	1–100 *μ*M	↓TG, ADRP, and SREBP-1	Chu et al. (2011) [75]
	High fat/cholesterol/bile salt-fed male ICR mice (for 7 days)	50–200 mg/kg/d × 6 doses p.o.	↓hepatic TC and TG	Pan et al. (2008) [76]
	Male ICR mice	0.2–1.6 g/kg × 1 dose p.o.	↑serum/hepatic TG, hepatic index; ↓hepatic TC and no changes in ALT & AST	Pan et al. (2011) [77]
	Male ICR mice (Sch B in combination with cholesterol/bile salt (2/0.5 g/kg))	1 g/kg × 1 dose	↑serum/hepatic TG, serum TC, and serum ALT and AST	Pan et al. (2013) [78]
	High fat diet-fed male C57BL/6 mice (for 20 days)	50 mg/kg/d × 14 doses	↓hepatic TG and palmitic acid; no changes in plasma TC	Kwan et al. (2015) [79]
	24 h fasting male C57BL/6 mice	0.8 g/kg × 1 dose	↑plasma TG and TC	Kwan et al. (2015) [79]

Antioxidant effects against fibrosis	CCl_4_-induced hepatotoxicity in Balb/c mice	20 mg/kg × 15 doses p.o.	↓plasma SDH, hepatic mitochondrial MDA; ↑hepatic mitochondrial GSH	Leong et al. (2012) [80]

Anti-inflammatory activity	LPS-activated RAW264.7 macrophages	25–50 *μ*M	↓TNF-*α*, IL-6, IL-1*β*, and NO	Leong et al. (2016) [81]

Anti-ER stress actions	Palmitic acid-induced steatotic HepG2 hepatocytes	10–100 *μ*M	↓GRP78, CHOP, XBP1, and TG	Jang et al. (2016) [82]
	Tunicamycin-challenged C57BL/6 mice	1 and 30 mg/kg × 4 doses	↓GRP78, CHOP, XBP1, TC, and TG	Jang et al. (2016) [82]

TG: total triglyceride; ADRP: adipose differentiation-related protein; SREBP1: sterol regulatory element-binding protein-1; GSH: reduced glutathione; TC: total cholesterol; ALT: alanine transaminase; AST: aspartate aminotransferase; SDH: sorbitol dehydrogenase; MDA: malondialdehyde; Grp78: glucose-regulated protein-78; CHOP: CCAAT/enhancer binding protein homologous protein; XBP1: X-box-binding protein-1; CCl_4_: carbon tetrachloride.
